# Cystathionine-Gamma-Lyase-Derived Hydrogen Sulfide-Regulated Substance P Modulates Liver Sieve Fenestrations in Caecal Ligation and Puncture-Induced Sepsis

**DOI:** 10.3390/ijms20133191

**Published:** 2019-06-29

**Authors:** Ravinder R Gaddam, Stephen Chambers, Robin Fraser, Victoria C Cogger, David G Le Couteur, Isao Ishii, Madhav Bhatia

**Affiliations:** 1 Department of Pathology and Biomedical Science, University of Otago, Christchurch 8140, New Zealand; 2 Ageing and Alzheimers Institute, ANZAC Research Institute, University of Sydney and Concord Hospital, Sydney 2139, Australia; 3 Showa Pharmaceutical University, Machida 194-8543, Japan

**Keywords:** hydrogen sulfide, substance P, cystathionine-gamma-lyase, neurokinin-1 receptor, liver sieve

## Abstract

Cystathionine-γ-lyase (CSE) is a hydrogen sulfide (H_2_S)-synthesizing enzyme that promotes inflammation by upregulating H_2_S in sepsis. Liver sinusoidal endothelial cells (LSECs) are fenestrated endothelial cells (liver sieve) that undergo alteration during sepsis and H_2_S plays a role in this process. Substance P (SP) is encoded by the preprotachykinin A (PPTA) gene, and promotes inflammation in sepsis; however, its regulation by H_2_S is poorly understood. Furthermore, the interaction between H_2_S and SP in modulating LSEC fenestrations following sepsis remains unclear. This study aimed to investigate whether CSE/H_2_S regulates SP and the neurokinin-1 receptor (NK-1R) and modulates fenestrations in LSECs following caecal ligation and puncture (CLP)-induced sepsis. Here we report that the absence of either CSE or H_2_S protects against liver sieve defenestration and gaps formation in LSECs in sepsis by decreased SP-NK-1R signaling. Following sepsis, there is an increased expression of liver CSE and H_2_S synthesis, and plasma H_2_S levels, which were aligned with higher SP levels in the liver, lungs and plasma and NK-1R in the liver and lungs. The genetic deletion of CSE led to decreased sepsis-induced SP and NK-1R in the liver, lungs and plasma SP suggesting H_2_S synthesized through CSE regulates the SP-NK-1R pathway in sepsis. Further, mice deficient in the SP-encoding gene (PPTA) preserved sepsis-induced LSEC defenestration and gaps formation, as seen by maintenance of patent fenestrations and fewer gaps. In conclusion, CSE/H_2_S regulates SP-NK-1R and modulates LSEC fenestrations in sepsis.

## 1. Introduction

Hydrogen sulfide (H_2_S) is an endogenous gasotransmitter and plays a role in inflammation. H_2_S synthesized through cystathionine-γ-lyase (CSE), a predominant H_2_S-synthesizing enzyme in peripheral organs, promotes inflammation in various inflammatory conditions including sepsis [1]. Upregulation of CSE/H_2_S signaling promotes leukocyte infiltration and organ injury in sepsis [2,3,4]. H_2_S donors, such as sodium hydrosulfide (NaHS) and sodium sulfide (Na_2_S) promote liver and lung inflammation and systemic inflammatory response through ERK1/2-NF-κB and subsequent release of proinflammatory cytokines and chemokines [3,5,6]. In contrast, treatment with a CSE inhibitor (PAG) [5,6], silencing of the CSE gene with siRNA [4] and mice deficient in the CSE gene [7] conferred protection against H_2_S-mediated inflammatory response and organ injury in sepsis. These studies suggest that H_2_S has an important role in modulating the inflammatory process during sepsis.

Substance P (SP) is an 11 amino acid immunoregulatory neurokinin belonging to the tachykinin family and is encoded by the preprotachykinin A (PPTA) gene. Although SP is a peptide primarily of neuronal origin, its production from inflammatory cells such as macrophages, eosinophils and dendritic cells have been reported [8]. The physiological actions of SP are primarily regulated through the neurokinin-1 receptor (NK-1R) [9]. Activation of NK-1R by SP leads to an increased release of inflammatory mediators and stimulates the chemotaxis of immune cells such as neutrophils, lymphocytes and monocytes. SP is one of the critical inflammatory mediators in inflammatory disease including sepsis. Research from our laboratory and other studies have reported the proinflammatory role of SP in both experimental and clinical sepsis; these actions of SP in sepsis are primarily mediated through NK-1R [10,11].

The interaction between H_2_S and SP in regulating inflammation of sepsis is poorly understood. For example, the H_2_S donor NaHS increased SP, which in turn promoted inflammation and lung injury through NK-1R activation following the induction of sepsis [12]. The mechanism includes activation of the transient receptor potential vanilloid type 1 (TRPV1) and, subsequently, SP and ERK1/2-NF-κB, to promote systemic inflammation and multiple organ failure in sepsis [13,14]. This provides some evidence for the role of SP in H_2_S-induced inflammation during sepsis. However, these studies were limited by the lack of use of specific pharmacological inhibitors (e.g., CSE inhibitors such as PAG) or activators (e.g., H_2_S donors such as NaHS and Na_2_S) [2,3,5,6,13,14,15,16]. The gene deletion approach offers a more definitive method for studying the interaction between H_2_S and SP in sepsis.

The liver has specialized endothelial cells called liver sinusoidal endothelial cells (LSECs). These are fenestrated in nature (liver sieve) with pore size in the range of 50–250 nm in diameter. The liver sieve acts as a permeable barrier between sinusoidal blood and hepatic parenchyma [17] and plays an important role in maintaining normal homeostasis of solutes, fluids, particles and metabolites between sinusoidal blood and hepatic parenchyma [18]. Alterations in liver sieve fenestration are associated with different inflammatory conditions, including LPS-induced endotoxaemia [19,20] and CLP-induced sepsis [7]. A recent study from our laboratory showed that H_2_S is one of the key inflammatory mediators of LSEC damage during CLP-induced sepsis [7]. However, how H_2_S contributes to LSEC damage and multiple organ damage in sepsis remains unknown.

In the present study, mice deficient in the H_2_S-synthesizing enzyme (CSE) and SP encoding gene (PPTA) were used to determine whether CSE/H_2_SregulatesSP-NK-1Rsignaling and SP modulates LSEC fenestration in sepsis.

## 2. Results

### 2.1. CSE-derived H_2_S Regulates SP Following CLP-Induced Sepsis

**Figure 1:**
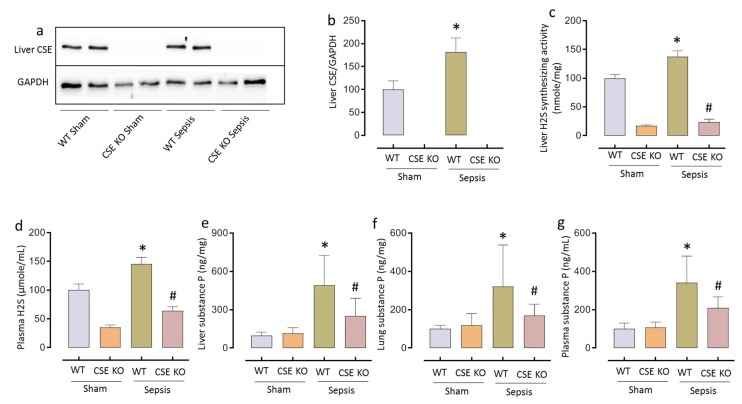
CSE-derived H_2_S regulates SP following CLP-induced sepsis. (**a**–**d**) Increased liver CSE expression (**a**) and quantification of CSE in ‘**a**’ (**b**), liver H_2_S-synthesizing activity (**c**) and plasma H_2_S levels (**d**) following CLP-induced sepsis. (**e–g**) Genetic deletion of CSE significantly decreased SP levels in liver (**e**), lung (**f**) and plasma (**g**) compared to WT mice following sepsis. Data represented as mean ± S.E.M. (*n* = 8). The significance of differences among groups was evaluated by ANOVA with post-hoc Tukey’s test. Statistical significance was assigned as *p* < 0.05 (* *p* < 0.05 vs. WT sham; # *p* < 0.05 vs. WT sepsis).

Sepsis induced by CLP in mice resulted in a significant increase in liver CSE expression, H_2_S-synthesizing activity and plasma H_2_S levels (* *p* < 0.05 vs. WT sham). Increased CSE/H_2_S signaling correlated with upregulated SP levels in the liver, lung, and plasma in sepsis (* *p* < 0.05 vs. WT sham). Mice deficient in the CSE gene had significantly lower levels of liver, lung and plasma SP compared to WT mice following sepsis(^#^ *p* < 0.05 vs. WT sepsis), suggesting that CSE/H_2_S acts as an upstream regulator of SP (Figure 1).

### 2.2. CSE-Derived H_2_S Regulates NK-1R Expression in Sepsis

**Figure 2:**
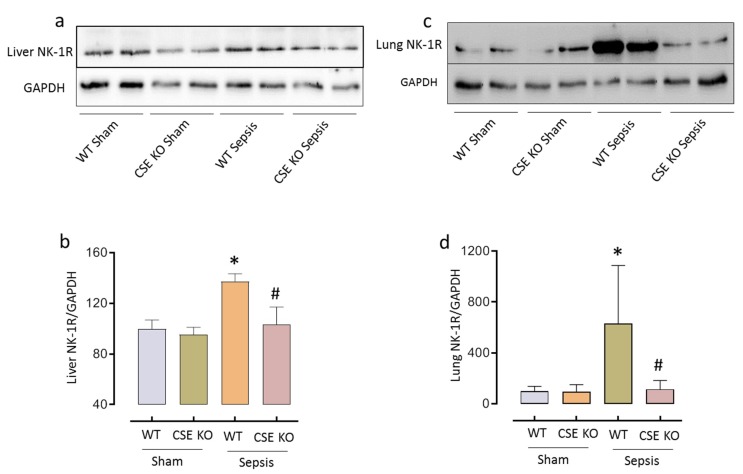
CSE-derived H_2_S regulates NK-1R following CLP-induced sepsis. (**a**–**d**) Genetic deletion of CSE significantly reduced liver (**a**) and lung (**c**) NK-1R protein expression compared to WT mice following sepsis and quantification of liver (**b**) and lung (**d**) NK-1R protein expression in ‘a’ and ‘c’, respectively. Data represented as mean ± S.E.M. (*n* = 8). The significance of differences among groups was evaluated by ANOVA with post-hoc Tukey’s test. Statistical significance was assigned as *p* < 0.05 (* *p* < 0.05 vs. WT sham; # *p* < 0.05 vs. WT sepsis).

Densitometry analysis of western blots showed that liver and lung NK-1R protein expression increased significantly following sepsis (* *p* < 0.05 vs. WT sham). Decreased H_2_S levels following CSE gene deletion significantly decreased sepsis-induced NK-1R expression (^#^ *p* < 0.05 vs. WT sepsis) (Figure 2).

### 2.3. PPTA Gene Deletion Protects Against Sepsis-Induced Damage to LSECs

**Figure 3:**
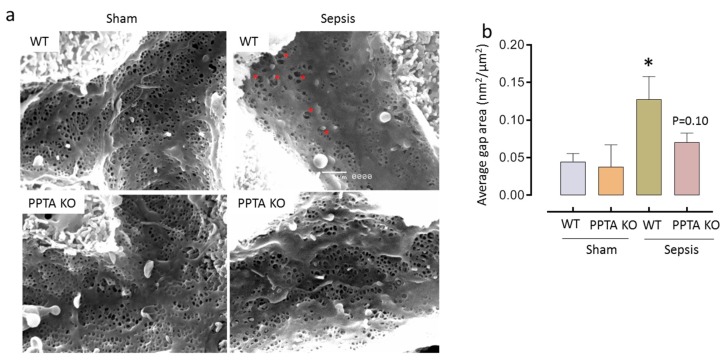
PPTA KO mice protect against sepsis-induced LSEC defenestration and gaps formation. (**a**,**b**) Representative images of liver sieve fenestration micrographs (**a**) and average gap area of liver sieve fenestrae (**b**). LSEC injury significantly increased (as evidenced by gaps formation) following CLP-induced sepsis in WT mice. Genetic deletion of PPTA in mice showed fewer gaps following sepsis compared to WT sepsis mice. Data represented as mean ± S.E.M. (*n* = 4). The significance of differences among groups was evaluated by ANOVA with post-hoc Tukey’s test. Statistical significance was assigned as *p* < 0.05 (* *p* < 0.05 vs. WT sham; # *p* < 0.05 vs. WT sepsis).

Scanning electron micrographs showed that CLP-induced sepsis promote decrease in LSEC fenestration frequency and porosity and increased gaps formation, compared to WT sham (gap area: 0.12 ± 0.02 nm^2^/mm^2^ vs. 0.06 ± 0.01 nm^2^/mm^2^; * *p* < 0.05 vs. WT sham). Mice, deficient in the PPTA gene had preserved LSEC morphology (fenestration frequency and porosity) and fewer gaps with sepsis, than WT with sepsis (gap area: 0.09 ± 0.01 nm^2^/mm^2^ vs. 0.12 ± 0.02 nm^2^/mm^2^) (Figure 3 and Table 1).

## 3. Discussion

This is the first study demonstrating that H_2_S synthesized through CSE enzyme (using CSE deficient mice) regulates SP and NK-1R, suggesting that SP plays a key role in promoting H_2_S-mediated LSEC damage in sepsis.

The proinflammatory role of SP and NK-1R has already been reported in sepsis and endotoxaemia-associated organ injury [9,10,21,22,23,24]; however, the mechanisms that regulating SP activation remain unclear. In the present study, a significant decrease in plasma and tissue (liver and lung) SP levels and subsequent NK-1R expression (in liver and lung) following sepsis in CSE knockout mice suggests CSE/H_2_S is one of the key mechanisms regulating SP and NK-1R in sepsis. The findings of the present study are consistent with earlier observations made using a pharmacological inhibitors of CSE [13,14,16] and H_2_S donors [12]; these results reinforce the essential role of CSE-derived H_2_S on SP-mediated inflammation and organ injury in sepsis.

Previous clinical studies of patients with sepsis and septic shock have shown higher circulating levels of SP [23]. To support this, a recent study from our laboratory showed that patients admitted to the ICU with sepsis had higher circulating SP levels than controls. These preceded elevated circulatory H_2_S levels, suggesting that H_2_S is an upstream regulator of SP [11]. The data in the present study strongly correlates with results from studies in clinical sepsis of H_2_S and SP, and suggests that both of these mediators serve as possible early stage diagnostic inflammatory markers in patients with sepsis.

The present study showed that a sepsis-induced an increase in SP and promotes defenestration and gap formation in LSECs. Endotoxaemia and the sepsis-induced inflammatory response are known to regulate the structure of LSEC fenestrations, i.e., defenestration in LSECs [20,25,26,27,28]. For example, mice challenged with pyocyanin (a virulence factor of *Pseudomonas aeruginosa)* and endotoxaemia (induced by LPS) promote liver injury associated with LSEC defenestration [29,30]. Further, in vitro exposure of LSECs to LPS or pyocyanin leads to changes in LSECs fenestration and disruption of liver sieve plates [29,31]. A recent study showed that decreased CSE/H_2_S signaling protects against LSEC defenestration and gaps formation following CLP-induced sepsis [7]. The results of the current study confirm that septic mice deficient in the PPTA gene (SP encoding gene) have less defenestration and fewer gaps formation in LSEC fenestrae than controls, and suggesting that CSE/H_2_S signaling occurs upstream to SP and regulates liver sieve fenestration.

Structural integrity of the fenestrated liver sinusoidal endothelium is believed to be essential for the normal homeostasis of fluids, particles and metabolites between hepatocytes and sinusoidal blood. Its alteration can have adverse effects on liver function. It is known that severe sepsis may lead to dysregulation of the LSEC [32]. The results of the present study suggest that H_2_S-regulated SP is one of the key proinflammatory mediators in modulating the LSEC phenotype and therefore that pharmacological manipulation of the liver sieve through H_2_S/SP plays an important role in the management of sepsis-associated fibrosis and cirrhosis.

Although the present study shows that H_2_S is one of the key mechanisms regulating SP in sepsis and the associated liver sieve phenotype, this is not a solitary mechanism. Mice deficient in the CSE gene have significantly reduced SP levels following sepsis; however, not to the extent of the control groups. This indicates there are other mechanisms through which SP is regulated in sepsis. During sepsis, an increased activation of immune cells such as macrophages plays an important role in liver sieve defenestration. Previous studies have suggested that an increased release of proinflammatory mediators, particularly TNF-α and biologically-active free radicals such as oxygen and nitrogen radicals lead to significant LSEC damage and that Kupffer cells, the resident macrophages in the liver, play a key role in this process [33]. Recent evidence has shown that Kupffer cells potentiate LSEC injury by ligating programmed death ligand-1 (PD-L1) in sepsis [34]. Since SP is known to be produced by macrophages, it is possible that activation of systemic and resident (Kupffer cells) macrophages leads to increased SP production and liver sieve defenestration. However, the precise mechanism of this interaction is not yet known.

In conclusion, the current study shows that SP, regulated by H_2_S, is a key inflammatory mediator that promotes liver sieve disruption in sepsis.

## 4. Materials and Methods

### 4.1. Induction of Sepsis

Both WT (C57BL/6J and BALAB/c) and KO (CSE KO on C57BL/6J background and PPTA KO on BALB/c background) mice were obtained from and maintained at Christchurch Animal Research Area (CARA). The CSE KO mice were generated as described previously [35]. All mice (male, aged 8–10 weeks) were randomly assigned into control (sham) and experimental groups (sepsis). For the first set of experiments, we used WT (C57BL/6J) and CSE KO mice (*n* = 8 for each group); in the second experiment, WT (BALB/c) and PPTA KO mice were used (*n* = 4 for each group). All experiments were approved (protocol number: C2/16 and approval on 16 May 2016) by the University of Otago-Christchurch Animal Ethics Committee and performed according to the established guidelines.

CLP-induced sepsis was performed according to the previously described protocol with minor modifications [3,27,36,37]. Briefly, buprenorphine (0.2 mg/kg) was injected subcutaneously 45 min before surgery (sham or CLP) for analgesia. Mice were anaesthetized by inhaled isoflurane (2%, 1 L/min O_2_). The caecum was exposed by a small midline incision through the skin and peritoneum of the abdomen (under sterile conditions). With silkam 5.0 thread, the caecal appendage was ligated at the distal part of the caecum (8–10 mm from the tip) then perforated using a 22-gauge needle. A small amount of stool was squeezed through each hole and the bowel was repositioned. The abdomen was sutured using sterile permilene 5.0 thread. Sham mice went through the same protocol but with no bowel perforation. In the first experiment, mice were euthanized 8 h after surgery by IP injection of pentobarbital sodium (150 mg/kg) and organs and plasma were collected and stored at −80 °C for subsequent experiments. In the second experiment, 8 h after surgery, mice were anaesthetized and livers were perfusion fixed with 2.5% glutaraldehyde in 0.1 M sodium cacodylate buffer to measure LSEC fenestrations using SEM as described previously [38].

### 4.2. H_2_S-Synthesizing Activity Assay

Liver H_2_S-synthesizing activity was measured as described previously [39]. Briefly, the liver samples were homogenized in PBS (20 mM, pH 7.4) with protease inhibitors. To measure H_2_S-synthesizing activity, 230 µL of homogenate was added to the reaction mixture containing L-cysteine (10 µL, 250 mM), and pyridoxyal5′-phosphate (10 µL, 18 mM). Samples were incubated for 30 min at 37 °C in a shaking water bath. Zinc acetate (1% wt/vol, 125 µL) was added to the tubes to trap evolved H_2_S. Next, a mixture of equal amounts of FeCl_3_ (30 µM) and N, N-dimethyl-p-phenylenediamine sulfate (20 µM) (133 µL, in 1:1 ratio) was added. Samples were incubated in the dark for 20 min and the reaction was stopped by the addition of 10% (*v*/*v*) trichloroacetic acid (25 µL). Samples were centrifuged and the resultant supernatant was used to measure the H_2_S concentrations using a spectrophotometer at 670 nm. Results were calculated against a Na_2_S calibration curve and corrected by protein content. Results were expressed as nmol H_2_S formed/mg protein.

### 4.3. Measurement of H_2_S Levels in Plasma

Plasma H_2_S levels were measured with a modified protocol based on methods described previously [39]. Briefly, the reaction mixture contained plasma (100 µL) in 20 mM sodium phosphate buffer (100 µL, pH 8.5), 1% (*w*/*v*) zinc acetate (100 µL), a mixture of N, N-dimethyl-p-phenylenediamine sulfate (20 µM) in 7.2 M HCl and FeCl_3_ (30 µM) in 1.2 M HCl (80 µL, in 1:1 ratio) and left to incubate at room temperature in the dark for 20 min. Following this, 10% (*v*/*v*) trichloroacetic acid (120 µL) was added to denature the protein and stop the reaction. Samples were centrifuged at 7700× *g* for 5 min and the absorbance of supernatant (150 µL) was measured with a 96-well microplate spectrophotometer at 670 nm. The H_2_S concentration was calculated against a calibration curve of Na_2_S. Results were expressed as µmole/mL.

### 4.4. Measurement of Substance P Levels

Liver and lung samples were homogenized in 1 mL of ice-cold sodium phosphate buffer for 20 s. The homogenates were centrifuged (13,000× *g*, 20 min, 4 °C) and the supernatants were collected. These were adsorbed on C18 cartridge columns (Bachem; Peninsula Laboratories, Bubendorf, Switzerland) as described [24,40]. The adsorbed peptide was eluted with 1.5 mL of 75% (*v*/*v*) acetonitrile. The samples were freeze-dried and reconstituted in SP assay buffer. The levels of SP were determined using ELISA (Bachem; Peninsula Laboratories, Bubendorf, Switzerland) according to the manufacturer’s instructions. The results obtained were then corrected for the protein content of tissue samples and expressed as nanograms per milligram of protein or nanograms per 100 µL of plasma.

### 4.5. Western Blotting

Tissue lysates (liver and lung) were prepared in ice-cold RIPA buffer supplemented with protease inhibitor cocktail by homogenization. The tissue homogenates were kept on ice for 30 min, followed by centrifugation at 10,000× *g* for 10 min. Protein samples were prepared and run through 10% SDS-PAGE gel for separation. Gels were transferred onto a nitrocellulose membrane (0.45 µm) and membranes blocked with 5% BSA for 1 h, followed by incubation with the primary antibody for CSE and NK-1R (1:1000) at 4 °C overnight, then by 2 h incubation with the secondary antibody (1:10,000) at room temperature before detection with a chemiluminescent substrate (Supersignal West Pico, Thermo Scientific Pierce Protein Biology, (Grand Island, NY, USA). Detection and quantification were performed on a chemi-doc system (Uvitec, Cambridge, UK). Mouse anti-human CSE was purchased from Abnova (Taipei City, Taiwan) and mouse anti-mouse NK-1R antibody was purchased from Abcam (Victoria, Australia). Rabbit anti-mouse glyceraldehyde-3-phosphate dehydrogenase (GAPDH) and goat anti-rabbit horseradish peroxidase (HRP)-conjugated antibodies were purchased from Santa Cruz Biotechnology (Santa Cruz, CA, USA).

### 4.6. Scanning Electron Microscopy (SEM)

To assess the liver sieve fenestrations using SEM, liver samples were perfusion fixed with phosphate buffered saline followed by paraformaldehyde. Perfusion fixed samples were cut into small blocks and processed (using 0.1 M cacodylate buffer/2% sucrose, 1% tannic acid/2% sucrose, 1% osmium/2% sucrose pH 7.4, and a series of ethanol (50%, 70%, 90%, 95%, and 100% and) with hexamethyldisilazane (HMDS)) for SEM. The liver blocks were mounted on SEM slug mounts and a platinum coat was applied using a sputter coater. Using SEM (JSM6380 SEM (JEOL, Japan), at 15 kV acceleration voltage, the blocks were examined. The fenestration diameter, frequency and total area were assessed using Image J (NIH) and porosity and number of gaps (pores greater than 250 nm in diameter, considered to represent non-specific LSEC damage) were calculated [38].

### 4.7. Statistical Analysis

Data are expressed as mean ± standard error of mean. One-way ANOVA with post-hoc Tukey’s test was performed to compare multiple groups using GraphPad version 6.07. *p* < 0.05 was considered statistically significant.

## Figures and Tables

**Table 1:** PPTA KO mice protect against sepsis-induced LSEC defenestration and gaps formation.

Group	Diameter of Fenestrae (nm)	Number of Fenestrae/µm^2^	Porosity (%)
**WT Sham**	149.39 ± 12.40	10.63 ± 1.54	18.44 ± 2.18
**PPTA KO Sham**	135.83 ± 7.83	11.60 ± 2.56	17.64 ± 3.28
**WT Sepsis**	143.91 ± 9.04	6.40 ± 2.49 *	11.05 ± 3.23 *
**PPTA KO Sepsis**	144.68 ± 8.39	9.05 ± 0.94	15.71 ± 2.19

The liver sieve frequency and porosity was lower in septic WT mice, and native fenestration frequency and porosity in LSECs was preserved in mice with genetic deletion of PPTA. Data represented as mean ± S.E.M. (*n* = 4). The significance of differences among groups was evaluated by ANOVA with post-hoc Tukey’s test. Statistical significance was assigned as *p* < 0.05 (* *p* < 0.05 vs. WT sham).
